# Evaluation of Novel Obesity and Lipid-Related Indices as Indicators for the Diagnosis of Metabolic Syndrome and Premetabolic Syndrome in Chinese Women with Polycystic Ovary Syndrome

**DOI:** 10.1155/2021/7172388

**Published:** 2021-08-17

**Authors:** Qianqian Yin, Jianhua Zheng, Yijuan Cao, Xiaonan Yan, Hong Zhang

**Affiliations:** ^1^Department of Obstetrics and Gynecology, The Second Affiliated Hospital of Soochow University, No. 1055, Sanxiang Road, Suzhou, Jiangsu 215000, China; ^2^Center for Reproductive Medicine, XuZhou Central Hospital, No. 199, Jiefang Road, Xuzhou, Jiangsu 221009, China; ^3^Department of Obstetrics and Gynecology, XuZhou Central Hospital, No. 199, Jiefang Road, Xuzhou, Jiangsu 221009, China

## Abstract

**Objectives:**

According to the International Diabetes Federation (IDF) criteria, previous studies in Chinese women with polycystic ovary syndrome (PCOS) reported a low prevalence of metabolic syndrome (MS); however, the same population predisposed to developing pre-MS. Early identification and treatment of individuals with MS and pre-MS are imperative to prevent their adverse consequences. Moreover, fasting plasma glucose (FPG) was not accurate in detecting pathoglycemia in women with PCOS as they have shown characteristically postprandial abnormalities in the carbohydrate metabolism. Therefore, we aimed to compare the discriminative performance of various indices for identifying MS and pre-MS/MS (pre-MS and MS) using the updated Chinese Diabetes Society (uCDS) criteria in Chinese women with PCOS.

**Methods:**

1083 Chinese women with PCOS were included in this study. We measured and evaluated 8 indices in all individuals. Based on the uCDS criteria for MS, patients who had no less than two components of MS but did not meet the criteria for the diagnosis of MS were considered as having pre-MS. Receiver operating characteristic (ROC) curves and the area under ROC curves (AUCs) levels were used to assess the accuracy of each index in detecting MS and pre-MS/MS.

**Results:**

Among the 8 indices assessed, the lipid accumulation product (LAP) provided the highest AUCs for detecting MS and pre-MS/MS, followed by CVAI, WTI, VAI, TyG, TG/HDL, WC, and BMI. The optimal cutoff points determined for LAP were 45.13 (sensitivity 88.0%, specificity 88.4%, and Youden index 0.764) for MS and 28.01 (sensitivity 87.5%, specificity 80.7%, and Youden index 0.681) for pre-MS/MS, respectively.

**Conclusions:**

uCDS criteria are reasonably more suitable for detecting MS and pre-MS in Chinese women with PCOS. Based on this criterion, LAP is the best index for the diagnosis of MS and pre-MS/MS in Chinese women with PCOS, out of the 8 obesity and lipid-related indices assessed.

## 1. Introduction

Metabolic syndrome (MS) is a cluster of metabolic abnormalities characterized as abdominal obesity, pathoglycemia, dyslipidemia, and hypertension [1]. Patients with MS are at an increased risk of suffering from type 2 diabetes mellitus (T2DM), cardiovascular disease (CVD), and several cancers [1, 2]. Polycystic ovary syndrome (PCOS), a widespread endocrine disorder, impacts 5–10% of women in their reproductive age [2]. Women with PCOS of different races are at increased risk of having MS [2, 3]. However, according to the International Diabetes Federation (IDF) criteria, previous studies in Chinese women with PCOS reported a low prevalence of MS; however, the same population showed a high occurrence of individual metabolic disorders [2]. In other words, according to the IDF criteria, many Chinese women with PCOS are predisposed to developing pre-MS. They do not meet the criteria for the diagnosis of MS but do suffer from at least two components of MS [4]. Patients with pre-MS are also at high risk of suffering from T2DM and CVD [5]. Concerning the long-term health risks of these conditions, early identification and treatment of individuals with MS and pre-MS are imperative to prevent their adverse consequences in Chinese women with PCOS.

For the diagnosis of MS in women with PCOS, the most commonly used criteria were the IDF criteria and the National Cholesterol Education Program Adult Treatment Panel III (ATP III) criteria [6]. In both of these criteria, pathoglycemia is diagnosed using fasting plasma glucose (FPG) levels alone. Given the fact that abnormalities in the carbohydrate metabolism in women with PCOS are characteristically postprandial, FPG was not accurate in predicting dysglycemia in women with PCOS [7]. Therefore, the criterion for pathoglycemia of the updated Chinese Diabetes Society (uCDS) criteria is reasonably more suitable for women with PCOS. Compared to both IDF and ATP III criteria, pathoglycemia in the uCDS criteria also includes patients with 2 h plasma glucose ≥7.8 mmol/L diagnosed using the oral glucose tolerance test (OGTT), except for FPG ≥ 6.1 mmol/L [3].

Several scientific organizations recommend periodic screening of PCOS patients to detect early metabolic disorders [8]. Thus, we need periodic screening of all five components of MS in each individual. In addition, according to the uCDS criteria, the OGTT should be performed in each individual. However, this process is unsuitable for mass screening because it is inconvenient and time-consuming. Therefore, it is crucial to identify simple, economical, and accurate indicators for the diagnosis of MS and pre-MS in Chinese women with PCOS.

Central obesity and visceral fat are major risk factors for MS. Therefore, in general populations, a series of studies focused on various obesity and lipid-related indices for detecting MS—lipid accumulation product (LAP) [9–13], visceral adiposity index (VAI) [9–13], Chinese visceral adiposity index (CVAI) [14], waist circumference-triglyceride index (WTI) [15], triglyceride (TG)-to-HDL-c ratio (TG/HDL) [9, 10, 16], and triglyceride glucose index (TyG) [17].

To our knowledge, women with PCOS show more visceral fat accumulation than those without PCOS [18]. Therefore, the aforementioned indicators may have different implications for the screening of MS among the PCOS population than in the general population. However, few studies have applied these indices for identifying MS in women with PCOS. Furthermore, these studies researched only a limited number of indices [19–23]; moreover, no group studied these indices for identifying pre-MS in women with PCOS. Thus, we conducted a cross-sectional study with Chinese PCOS patients to reveal the prevalence of metabolic disorders using the uCDS criteria. We also compared the discriminative performance of these indices for identifying women with MS and pre-MS.

## 2. Materials and Methods

### 2.1. Subjects and Study Design

A total of 1083 women with PCOS, aged 16–41 years, were recruited between July 2012 and December 2020, at the Center for Reproductive Medicine and the Gynecological Outpatient Department of Xuzhou Central Hospital. Any medications known to affect sex hormone and glucose or lipid metabolism were discontinued for at least three months before the study. The subjects were not allowed to use any antihypertensive drugs. The study was approved by the Institutional Review Board of Xuzhou Central Hospital. Prior informed consent was obtained from either a legal guardian of each patient younger than 18 years old or from those subjects who were ≥18 years.

Patients were diagnosed with PCOS according to the Rotterdam criteria [2], if they met at least two of the following three criteria: (i) oligo/anovulation—menstrual cycle ＞35 days in length or ≤ 8 menstrual periods in a year), (ii) clinical or biochemical hyperandrogenism—acne or modified Ferriman–Gallwey scores ≥ 6 or serum testosterone (T) ≥ 2.6 nmol/L or free testosterone (FT) ≥ 9.0 pg/mL, and (iii) polycystic morphology of ovaries in ultrasonography. Additionally, the exclusion criteria consisted of related disorders, such as hypothyroidism, hyperprolactinemia, and adrenal hyperplasia.

uCDS criterion was confirmed when at least three of the following five criteria were met [3]: (i) waist circumference (WC) ≥ 85 cm, (ii) TG concentration ≥ 1.7 mmol/L, (iii) HDL-c concentration < 1.0 mmol/L, (iv) systolic blood pressure (SBP) ≥ 130 mmHg or diastolic blood pressure (DBP) ≥ 85 mmHg or previously diagnosed high blood pressure (HBP), and (v) FPG ≥ 6.1 mmol/L or 2 h plasma glucose ≥ 7.8 mmol/L or previously diagnosed T2DM.

Pre-MS is defined as having no less than two components of MS, but not meeting the criteria for the diagnosis of MS [4].

### 2.2. Study Protocol

All patients underwent a 75 g OGTT and anthropometric measurements. All parameters were measured as previously described [5].

Fasting blood samples were obtained between the first and fifth day of the menstrual period/withdrawal bleeding. Prolactin, T, FT, and thyroid-stimulating hormone (TSH) were assessed by chemiluminescence immunometric assay (Beckman Unicel DxI 800; Snibe MAGLUMI 4000; Abbott Immulite 2000 analyzer). 17-*α*-Hydroxyprogesterone was measured using the enzyme-linked immunosorbent assay (ELISA). Plasma glucose was measured using the glucose oxidase method (Hitachi 7600 autoanalyzer). Plasma insulin was measured using a chemiluminescence immunometric assay (Roche e601 analyzer). Total cholesterol (CHOL), TG, HDL-c, and low-density lipoprotein cholesterol (LDL-c) were measured using the enzymatic colorimetric method (Hitachi 7600 autoanalyzer). Indices were calculated based on the following formulas.

  Body mass index (BMI) = weight (kg)/height^2^ (m)
  LAP = (WC (cm)–58) × TG (mmol/L) [9].
  CVAI = –187.32 + 1.71 × age + 4.23 × BMI + 1.12 × WC (cm) + 39.76 × log10[TG (mmol/L) ] – 11.66 × HDL (mmol/L) [14].
  VAI = [WC (cm)/[36.58 + (1.89 × BMI)]] × [TG (mmol/L)/0.81] × [1.52/HDL-C (mmol/L)] [15].
  WTI = WC (cm) × TG (mmol/L) [15].
  TyG = Ln (fasting triglycerides (mg/dL) × fasting glucose (mg/dL)/2) [17].

### 2.3. Statistical Analysis

The data were analyzed using the statistical software, SPSS version 24.0 for Windows. Continuous variables were described as median with 25^th^–75^th^ percentiles, and the difference between the groups was determined using the Mann–Whitney *U* test. Receiver operating characteristic (ROC) curves and the area under ROC curves (AUC) were determined to assess the accuracy of each index in detecting AGT. Differences in AUC were evaluated using the Hanley and McNeil method. A *p* value of ≤0.05 (two-tailed) was considered statistically significant.

## 3. Results

Out of the 1083 subjects, 4.71% (*n* = 51) were diagnosed with T2DM, 4.52% (*n* = 49) had FPG ≥ 6.1 mmol/L, 16.6% (*n* = 180) had FPG ≥ 5.6 mmol/L, and 21.98% (*n* = 238) had 2 h plasma glucose ≥ 7.8 mmol/L. Of the 238 subjects with 2 h plasma glucose ≥7.8 mmol/L, 63.03% (*n* = 150) had FPG < 5.6 mmol/L and 86.13% (*n* = 205) had FPG < 6.1 mmol/L.

Table 1 summarizes the prevalence of MS, pre-MS, and MS components in Chinese women with PCOS using uCDS criteria.

The AUCs of all indices in detecting MS and pre-MS/MS (pre-MS and MS) were different by 0.5 (*p* < 0.05). Among all 8 indices, LAP showed the most diagnostic accuracy for MS and pre-MS/MS based on the highest AUC values—0.947 for MS and 0.912 for pre-MS/MS. This was followed by CVAI, WTI, VAI, TyG, TG/HDL, WC, and BMI. LAP displayed higher AUC values than the other indices (*p* < 0.05), except for CVAI (*p* > 0.05) (Table 2).

According to the uCDS criteria, the optimal cutoff points determined for LAP were 45.13 (sensitivity 88.0%, specificity 88.4%, and Youden index 0.764) for MS and 28.01 (sensitivity 87.5%, specificity 80.7%, and Youden index 0.681) for pre-MS/MS, respectively (Table 3).

**Figure 1:**
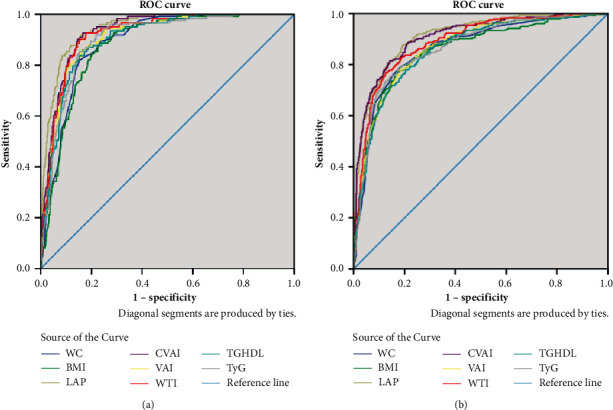
Receiver operating characteristic (ROC) curves for each index for detecting MS (a) and pre-MS/MS based on the uCDS criteria (b).

ROC curves for all indices in detecting MS and pre-MS/MS using uCDS criteria are shown in Figure 1.

## 4. Discussion

Glucose intolerance has long been recognized as a major risk factor for T2DM [24]. In our study, 63.03% of the PCOS women with glucose intolerance had FPG <5.6 mmol/L and 86.13% had FPG <6.1 mmol/L. This result confirmed the importance of detecting MS and pre-MS using uCDS criteria in Chinese women with PCOS.

A total of 8 indices were evaluated in our study, and the analysis of the AUC revealed that all 8 indices were capable of discriminating the subjects with MS and pre-MS/MS.

Kahn was the first to introduce the LAP, a novel index based on a combination of WC and TG [25]. Various studies in general population have suggested that LAP is a good indicator of MS. Based on IDF criteria, two studies of Chinese middle-aged and elderly people demonstrated that LAP performed better than VAI, TyG, TG/HDL, and BMI in identifying MS [9, 17]. Based on three different definitions for MS, Nascimento–Ferreira et al. [11] suggested that LAP had higher prediction accuracy for MS than WC and BMI in undiagnosed Brazilian adults. However, few studies have applied LAP for identifying MS in women with PCOS. Based on the IDF criteria, in Chinese women with PCOS, LAP performed best for detecting MS, followed by WC and BMI [20]. The same results were reported in Caucasian women with PCOS [19]. However, both studies included limited sample size. Moreover, no data are currently available establishing any association between LAP and pre-MS. Our study is the first to demonstrate that LAP is the strongest predictor for identifying MS and pre-MS/MS, based on uCDS criteria in Chinese PCOS population.

VAI, developed to estimate visceral adipose function in Caucasians, is an index estimated with the use of both anthropometric (BMI and WC) and metabolic (TG and HDL-C) parameters [9]. However, compared to equivalent Caucasians, Asian subjects showed a greater proportion of body fat for a given BMI level [26] and are more prone to accumulate visceral adipose [27]. Indeed, VAI was poorly associated with adipose tissue function in Chinese [14]. It was not surprising since VAI was developed for Caucasians. In 2016, another index, called CVAI, was developed to estimate the visceral fat function in Chinese [14]. It is based on a combination of age and all parameters of VAI. Xia et al. [14] reported that CVAI was better in predicting MS compared with BMI, WC, and VAI in a Chinese population. This was the only report on the relationship between CVAI and MS. In the present study, CVAI was the second most accurate for the diagnosis of MS and pre-MS/MS. However, there were no significant differences in the AUCs values between LAP and CVAI for detecting MS or pre-MS/MS.

Overall, our study concluded that WTI, VAI, TG/HDL, and TyG were inferior indicators compared with LAP and CVAI. Nonetheless, these four indices showed a high capacity to discriminate for MS and pre-MS/MS, with AUCs >0.8. According to Hosmer and Lemeshow's standard, they were “excellent” and “outstanding” indicators—excellent if 0.8 ≤ AUC < 0.9 and outstanding if AUC ≥ 0.9 [28]. WTI, similar to LAP, is also a continuous scale of WC and TG. It had good discriminate ability for MS and pre-MS/MS, but was inferior to LAP in our study, coherent with previous results [15, 29]. LAP is calculated as (WC − 58) × TG, while WTI as WC × TG. According to the formula, WTI decreases the impact of TG. It may be the reason for lowering the prediction accuracy of WTI than LAP. In addition, compared to the other lipid-related indices, we observed lower accuracy of TyG and TG/HDL for MS and pre-MS/MS, in accordance with previous results [9, 17]. This is mainly because TyG only combined FPG and TG, and TG/HDL only combined TG and HDL-c. The absence of WC, a significant parameter for MS prediction, in TyG and TG/HDL calculation decreases their accuracy as predictors.

In our study, LAP was the best indicator of MS and pre-MS/MS based on uCDS criteria. We determined its optimal cutoff point. Based on the uCDS criteria, the LAP index cutoff points for MS and pre-MS were 45.13 and 28.01, respectively.

For comparison with different criteria and other reported studies, we also evaluated the accuracy of each index for MS and pre-MS/MS by both IDF and ATP III criteria, in the form of AUC values. In accordance with the results by uCDS criteria, LAP displayed the highest accuracy among the 8 indices based on both IDF and ATP III criteria (data not shown). However, using the same IDF criteria, the cutoff point of LAP for detecting MS differs from other similar studies, such as another study of Chinese women with PCOS (54.2) [20] and of Serbian women with PCOS (25.90) [19]. Differences in sample sizes and racial populations being assessed may have led to these discrepancies in the optimal cutoff points among the studies.

Despite these relevant findings, it is important to point out the limitations of our study. All subjects in our study were recruited from the Center for Reproductive Medicine and Gynecological Outpatient Department of Xuzhou Central Hospital, China. This population may not represent all Chinese women with PCOS, with a possibility of bias in the results.

## 5. Conclusions

The uCDS criteria are reasonably more suitable for detecting MS and pre-MS in Chinese women with PCOS. Based on this criterion, LAP is the best index for the diagnosis of MS and pre-MS/MS in Chinese women with PCOS, out of the 8 obesity and lipid-related indices assessed.

## Figures and Tables

**Table 1:** Prevalence of MS, pre-MS, and MS components in Chinese women with PCOS based on the uCDS criteria.

	Case (%)
Occurrence of each component of MS, *n* (%)	
Raised BP	176 (16.25%)
Increased TG	253 (23.36%)
Central obesity	248 (22.90%)
Reduced HDL-c	49 (4.52%)
Increased plasma glucose	254 (23.45%)
Number of components	
0	550 (50.78%)
1	255 (23.55%)
2	154 (14.22%)
3	82 (7.57%)
4	39 (3.60%)
5	3 (0.28%)
Prevalence of MS	124 (11.45%)
Prevalence of pre-MS	154 (14.22%)

BP, blood pressure; TG, triglycerides; HDL-c, high-density lipoprotein cholesterol.

**Table 2:** The AUCs of each index for detecting MS and pre-MS/MS based on the uCDS criteria in Chinese women with PCOS.

	MS	Pre-MS/MS
BMI	0.880 (0.855–0.906)	0.858 (0.831–0.884)
WC	0.891 (0.868–0.915)	0.865 (0.839–0.890)
LAP	0.946 (0.930–0.962)	0.912 (0.893–0.932)
CVAI	0.933 (0.916–0.950)	0.909 (0.888–0.929)
VAI	0.912 (0.890–0.935)	0.868 (0.844–0.892)
WTI	0.922 (0.901–0.943)	0.889 (0.867–0.911)
TyG	0.904 (0.880–0.928)	0.867 (0.842–0.892)
TG/HDL	0.903 (0.879–0.926)	0.861 (0.836–0.886)

BMI, body mass index; WC, waist circumference; LAP, lipid accumulation product; VAI, visceral adiposity index; CVAI, Chinese visceral adiposity index; WTI, waist circumference-triglyceride index; TyG, triglyceride glucose Index; TG/HDL, triglycerides/high-density lipoprotein cholesterol; MS, metabolic syndrome; LAP vs. CVAI, *p* > 0.05. LAP vs. each of other 13 indices, *p* < 0.05.

**Table 3:** Receiver operating characteristic (ROC) curves data for detecting MS and pre-MS/MS using LAP based on the uCDS criteria.

	AUC	Cutoff point	Sensitivity (%)	Specificity (%)	Youden index
MS	0.947	45.13	88.0	88.4	0.764
Pre-MS/MS	0.912	28.01	87.5	80.7	0.681

## Data Availability

The data used to support the findings of this study are available from the corresponding author upon request.
